# Spliceosomal Factor *SmF* Modulates Temperature‐Mediated Flower and Leaf Size Plasticity in *Arabidopsis thaliana*

**DOI:** 10.1111/pce.70358

**Published:** 2026-01-05

**Authors:** Gregory M. Andreou‐Huotari, Mikael Brosché, Jan Hoffmann, Zoran Nikoloski, Roosa A. E. Laitinen

**Affiliations:** ^1^ Organismal and Evolutionary Biology Research Programme, Viikki Plant Science Centre University of Helsinki Helsinki Finland; ^2^ Systems Biology and Mathematical Modelling Max Planck Institute of Molecular Plant Physiology Potsdam Germany; ^3^ Bioinformatics Department, Institute of Biochemistry and Biology University of Potsdam Potsdam Germany

## Abstract

This study demonstrates that a core spliceosomal component, Smith antigen F (SmF) protein, is involved in regulating flower and leaf size plasticity in response to temperature and light, providing insight into the role of post‐transcriptional regulation in plant phenotypic plasticity.

1

Fluctuations in light and temperature are known to modulate plasticity in plant growth and development (Delker et al. 2022), and to influence alternative splicing in leaves (Hartmann et al. 2016; Capovilla et al. 2018). Yet, the role of alternative splicing in light‐ and temperature‐mediated trait plasticity remains elusive. Therefore, we grew 17 green fluorescent protein (GFP) reporter line mutants for 12 spliceosomal‐related genes (Kanno et al. 2016; Kanno et al. 2017; Kanno et al. 2020) under constant 17°C and 25°C and measured flower diameter (FD) (Tables S1 and S3). For quantification and biological interpretation, FD plasticity for each of the lines was calculated as a percentage of difference in FD from 17°C to 25°C (Figure 1A). Three mutants, namely: *cbp80‐1*, *hgf4‐1*, and *rbm25‐3*, showed significantly different FD plasticity compared to WT (Figure S1A). From these, *hgf4‐1* showed the largest reduction (−25%) of plasticity in comparison to WT, despite no differences in flowering time (Figure S2). The reduction of temperature‐mediated FD plasticity in *hgf4‐1* in comparison to WT was validated altogether in six independent growth trials (Figure S1B).

**Figure 1:**
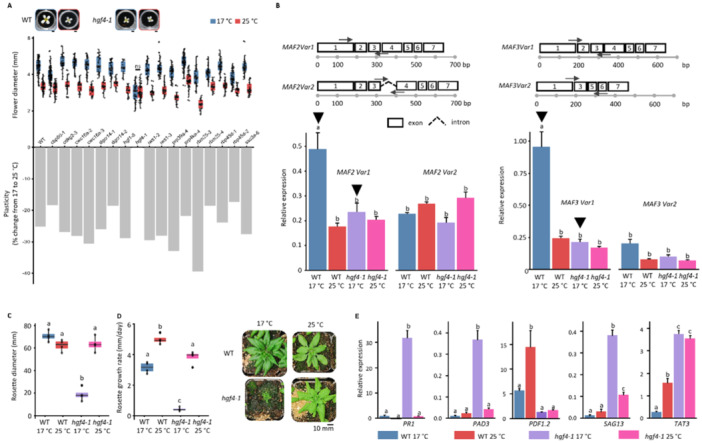
Characterization of temperature‐mediated flower and leaf size plasticities in *hgf4‐1* mutant. (A) Flower diameters of 17 spliceosomal‐related mutants (Table S1), and the resulting FD plasticity, from plants grown at 17°C and 25°C. Unpaired *t*‐test with Bonferroni correction showed mean flower diameters across the temperatures were significantly different for all lines, except *hgf4‐1* (*p*‐value < 0.001, ns= non‐significant). Only non‐significant (ns) differences are indicated. (B) *MAF2* and *MAF3* splice variants 1 and 2 were quantified via real time qRT‐PCR using *YLS8* and *TIP41‐like* for normalization via the delta delta Ct (amplification efficiency^‐ΔΔCt^) method. Error bars show standard error. (C) The maximum rosette size, and (D) rosette growth rate (RGR) of WT and *hgf4‐1* plants grown at 17°C and 25°C. RGR for WT and *hgf4‐1* was calculated as the slope of a linear function of rosette sizes scored from photographs taken over development. *n* = 4. (E) Gene expression profiling of defense genes in leaves of WT and *hgf4‐1* grown at constant 17 or 25°C using qRT‐PCR as before. Error bars show standard error. In B‐E: Tukey's HSD, Bonferroni corrected *p*‐value < 0.05. Groups that share a letter are not significantly different.

*hgf4‐1* carries a non‐synonymous substitution (P16L) at a predicted alpha‐helix hinge amino acid residue in *AT4G30220*, encoding for a Smith Antigen F (SmF) protein (Figure S3A) (Kanno et al. 2017). The mutation in SmF has been previously confirmed by complementation test to restore the WT GFP phenotype (Kanno et al. 2017). We did not observe altered *SmF* expression between *hgf4‐1* and WT flowers at 17 or 25°C (Figure S4A,B), indicating that the effect of the *SmF* mutation in *hgf4‐1* occurs at the protein level. Alpha fold protein modelling (Figure S3B), and known X‐ray crystallography structures for Sm‐proteins (Kambach et al. 1999), showed us that the SmF protein assembles with six other Sm‐proteins around the uridyl‐rich Sm‐site present in four of the major spliceosomal small nuclear RNAs, *i.e*., U1, U2, U4 and U5 snRNAs (pTM = 0.67, ipTM = 0.77, Figure S3B). SmF was further predicted to have several residue contact points to two other Sm ring proteins, where the proline at position 16 was modelled to be in contact with three SmE residues (Figure S3C). Hence, protein function in *hgf4‐1* was predicted to be affected through altered heptamer ring interaction, with broader implications for pre‐mRNA processing and alternative splicing.

To investigate the factors that render smaller *hgf4‐1* flowers at 17°C compared to WT, which in turn reduces the amount of plasticity, we examined petal epidermal conical cells. In WT, conical cells, like FD, were significantly larger at 17°C compared to 25°C (Figure S5A). By contrast, in *hgf4‐1*, the average area of conical cells did not change in response to temperature and the average area of conical cells was not significantly different from WT at 17°C. Furthermore, our analysis showed 45% and 20% reduction in the number of conical cells per petal in *hgf4‐1* in comparison to WT petals harvested at 17°C and 25°C, respectively (Figure S5B). These findings imply that that the reduced FD in *hgf4‐1* in comparison to WT, particularly at 17°C, is due to reduced cell division in petals rather than a lack of cell expansion.

Alternative splicing of hundreds of genes was affected in *hgf4‐1* in comparison to WT (Kanno et al. 2020). One of the genes that showed differential alternative splicing pattern in *hgf4‐1* leaves in comparison to WT was *MADS AFFECTING FLOWERING 3* (*MAF3*). *MAF3* is part of the *MAF2‐5* gene cluster, which in addition to regulating temperature‐dependent flowering time in *A. thaliana*, has been shown to regulate temperature‐mediated FD plasticity in the same species (Wiszniewski et al. 2022). To investigate whether splicing of *MAF2* and *MAF3*, both showing temperature‐dependent alternative splicing in leaves, was affected in *hgf4‐1* flowers at 17°C and 25°C, we investigated the abundance of *MAF2* and *MAF3* splice‐variants. Semi‐quantitative PCR revealed that both *MAF2* and *MAF3* exhibited temperature‐dependent alternative splicing in flowers, and the number of splice variants was the same as in leaves (Figure S6A). Further analysis using real‐time quantitative PCR showed that for both *MAF2* and *MAF3* genes, splice variant 1, in which all introns are spliced out and all exons are retained, was significantly more abundant at 17°C in the WT in comparison to *hgf4‐1* at 17°C (Figure 1B). This finding provides support that the reduced temperature‐mediated FD plasticity in *hgf4‐1* mutants was due to alterations in SmF mediated alternative splicing of *MAF2* and *MAF3*.

Next, we asked whether temperature affects leaf size plasticity in *hgf4‐1* by investigating rosette diameter (RD) at the onset of flowering, and if this is related to flowering time and rosette growth rate (RGR). Unlike for FD, *hgf4‐1* showed significantly higher temperature‐mediated RD plasticity than WT, through consistently significantly smaller RD at 17°C (Figure 1C, Figure S1B). Although flowering time was comparable at both temperatures between *hgf4‐1* and WT (Figure S2), RGR was consistently significantly slower at the lower temperature in *hgf4‐1* in comparison to WT (Figure 1D, Figure S1D).

To dissect the factors underlying the reduced RD at 17°C, we quantified average pavement cell area and estimated total pavement cell number in WT and *hgf4‐1* leaves grown at 17°C and 25°C. The mean average area of leaf pavement cells in *hgf4‐1* was significantly smaller compared to the WT at both temperatures (Figure S5C). The total estimated number of pavement cells, per whole leaves of *hgf4‐1* grown at 17°C, was reduced compared to WT (Figure S5D), implying that the reduced leaf growth at the lower temperature is due to impaired cell division and expansion. In addition, *hgf4‐1* at 17°C produced significantly fewer seeds per plant and lower germination rates compared to WT (Figure S7A,B) demonstrating that compared to WT, *hgf4‐1* mutant has reduced fitness at 17°C.

The well‐described growth defense trade‐off in plants, with activated defense responses resulting in strongly impaired growth, led us to ask if the reduced RD and fitness, quantified as total seed number and seed germination, in *hgf4‐1* at 17°C was associated with the activation of immune responses. We analyzed the expression of five marker genes for different defense pathways: *PATHOGEN RESPONSE 1* (*PR1*), *PHYTOALEXIN DEFICIENT 3 (PAD3), PLANT DEFENSIN 1.2 (PDF1.2) SENESCENCE‐ASSOCIATED GENE 13 (SAG13)*, and *TYROSINE AMINOTRANSFERASE 3 (TAT3)* in newly emerging leaves of WT and *hgf4‐1* (Methods, Table S2). We found that the expression of *PR1* and *PAD3* were significantly higher in *hgf4‐1* leaves in comparison to WT, only at 17°C (Figure 1E), indicating that salicylic acid‐dependent defense signaling is activated at 17°C. Stomatal number is known to be connected with increased defense (Kemppinen et al. 2025). To test if the stomatal number is affected in *hgf4‐1* at 17°C, we quantified the stomatal number in WT and *hgf4‐1*. However, no difference in stomatal number between WT and *hgf4‐1* at either temperature was observed (Figure S5E). The increased transcript levels for *SAG13* and *TAT3* in *hgf4‐1* leaves at both temperatures in comparison to WT (Figure 1E) suggests that *hgf4‐1* has enhanced stress tolerance. This was further supported by testing oxidative stress tolerance using ozone treatment. While WT leaves showed increased cell death in response to ozone treatment, *hgf4‐1* was resistant (Figure S5F). To test if oxidate stress is due to more open stomata in *hgf4‐1*, we tested if the mutant leaves showed reduced water loss in comparison to WT. Intriguingly, we found significantly higher water loss in the leaves of *hgf4‐1* compared to WT (Figure S5F), suggesting that stomata were more open in *hgf4‐1*. Thus, indicating that *hgf4‐1* has increased oxidative stress resistance, independent of stomata.

Light and temperature signaling in plants are known to be highly related with shared physiological responses (Legris et al. 2017), but it is unclear whether light affects flower and leaf size plasticity through SmF. To address this, we first asked whether the temperature‐mediated differences in FD and RD plasticity observed in *hgf4‐1* relative to WT are also affected by light intensity and whether these effects relates to flowering time. We grew the *hgf4‐1* and WT plants at 17°C and 25°C under low light (LL, 45 μmol m^−2^ s^−1^), and compared them with plants grown under normal light (NL, 180 μmol m^−2^ s^−1^). In WT, flowers were smaller under LL in comparison to NL at both temperatures, implying that FD shows plasticity to light intensity and that this response is temperature‐independent (Figure S8A). We then tested whether other spliceosome‐related mutants show altered light‐mediated plasticity. In difference to all other spliceosomal mutants, *hgf4‐1* consistently displayed the largest significant reduction of FD plasticity compared to WT across the four light and temperature‐mediated plasticity comparisons considered (Figure S9A–C). In *hgf4‐1* leaves, lower temperature restricted growth more strongly than reduced light (Figure S8B). Flowering time did not differ between WT and *hgf4‐1* under any tested conditions (Figure S8D). To validate these trait plasticities genetically, we analysed F_1_ plants from the backcross between *hgf4‐1* and WT. All flower and leaf traits reverted to WT levels (Figure S8A–D), demonstrating that the altered temperature and light‐mediated flower and leaf size plasticities in *hgf4‐1* were recessive. Together, these results showed that *SmF* independently modulates flower and leaf size plasticity in response to not only to temperature, but also to light. Yet, further experiments will be required to uncover the mechanisms that differentiate these trait‐specific plastic responses.

In summary, our results highlight a key role for a spliceosomal component in modulating flower and leaf size plasticity in response to both temperature and light in *A. thaliana*. Yet, while flower size responses were robust across environments in the *hgf4‐1* mutant in comparison to WT, leaf growth was more sensitive to temperature than light. This organ‐specific regulation highlights the complexity of spliceosome‐mediated environmental plasticity. These findings, summarized in Figure S10, provide new insight into how plants balance growth, reproduction, and stress resilience in fluctuating environments.

## Conflicts of Interest

The authors declare no conflicts of interest.

## Supporting information


**Supporting Figure 1:** Comparison of temperature‐mediated *hgf4‐1* and WT plasticities across six trials. **Supporting Figure 2:** Photographs of WT and *hgf4‐1* plants grown at 17 and 25°C. **Supporting Figure 3:** *In silico* modelling of Sm proteins. **Supporting Figure 4:** *SmF* expression in WT and *hgf4‐1* plants grown at 17 and 25°C. **Supporting Figure 5:** Microscopic and physiological analysis of petal and leaf cells in WT and *hgf4‐1* plants grown at 17 and 25°C. **Supporting Figure 6:** Splice variants and gene expression profiling of *MAF2* and *MAF3*. **Supporting Figure 7:** Fitness estimates for WT and *hgf4‐1* plants. **Supporting Figure 8:** Characterization of light‐mediated flower size plasticity in *hgf4‐1* grown at 17 and 25°C. **Supporting Figure 9:** Mean flower diameters and flower size plasticities of 17 spliceosome‐related mutants in response to temperature and light‐intensity. **Supporting Figure 10:** Summary of the key traits in *SmF* mutant *hgf4‐1* that were associated with differences in temperature and light‐mediated growth plasticity.

**Table S1:** Mutant lines used in this study. **Table S2:** Flower diameters and statistical testing of plant lines grown at constant 17 or 25°C. Plant lines were compared to WT at each temperature and also comparisons were made within one plant line across the two temperatures. (*t*‐test with Bonferroni correction, ***p‐value < 0.001. ***p‐value 0.01, *p‐value < 0.05, ns = not significant.). **Table S3:** Primers for the gene targets used in this study.

Supplemental Materials and Methods.

## Data Availability

The data that support the findings of this study are available from the corresponding author upon reasonable request.
