# Supplementary material for: Spliceosomal Factor SmF Modulates Temperature‐Mediated Flower and Leaf Size Plasticity in Arabidopsis thaliana
Source: Plant Cell Environ. 2026 Jan 5;49(4):2024–7. doi: 10.1111/pce.70358 (PMC12976579; doi:10.1111/pce.70358)
Supplement: Supplementary file 1 — Supporting Figure 1: Comparison of temperature‐mediated hgf4‐1 and WT plasticities across six trials. Supporting Figure 2: Photographs of WT and hgf4‐1 plants grown at 17 and 25°C. Supporting Figure 3: In silico modelling of Sm proteins. Supporting Figure 4: SmF expression in WT and hgf4‐1 plants grown at 17 and 25°C. Supporting Figure 5: Microscopic and physiological analysis of petal and leaf cells in WT and hgf4‐1 plants grown at 17 and 25°C. Supporting Figure 6: Splice variants and gene expression profiling of MAF2 and MAF3. Supporting Figure 7: Fitness estimates for WT and hgf4‐1 plants. Supporting Figure 8: Characterization of light‐mediated flower size plasticity in hgf4‐1 grown at 17 and 25°C. Supporting Figure 9: Mean flower diameters and flower size plasticities of 17 spliceosome‐related mutants in response to temperature and light‐intensity. Supporting Figure 10: Summary of the key traits in SmF mutant hgf4‐1 that were associated with differences in temperature and light‐mediated growth plasticity. [file PCE-49-2024-s003.pdf]

Supporting Figures for

Spliceosomal factor *SmF* modulates temperature-mediated flower and leaf size plasticity in *Arabidopsis thaliana*

Gregory M. Andreou-Huotari[1], Mikael Brosché[1], Jan Hoffmann[1], Zoran Nikoloski[2,3] and Roosa A. E. Laitinen[1*]

[1] Organismal and Evolutionary Biology Research Programme, Viikki Plant Science Centre, University of Helsinki, 00790 Helsinki, Finland
[2] Systems Biology and Mathematical Modelling, Max Planck Institute of Molecular Plant Physiology, 14476 Potsdam, Germany
[3] Bioinformatics Department, Institute of Biochemistry and Biology, University of Potsdam, 14476 Potsdam, Germany

*Corresponding author: Roosa Laitinen, Organismal and Evolutionary Biology Research Programme, Viikki Plant Science Centre, PO Box 65, FIN-00014 University of Helsinki, Tel. +358 (0) 29 4157787, E-mail: Roosa.Laitinen@Helsinki.fi

Paste corresponding author name here
Email: Roosa.Laitinen@Helsinki.fi

This PDF file includes:

Figures S1 to S10

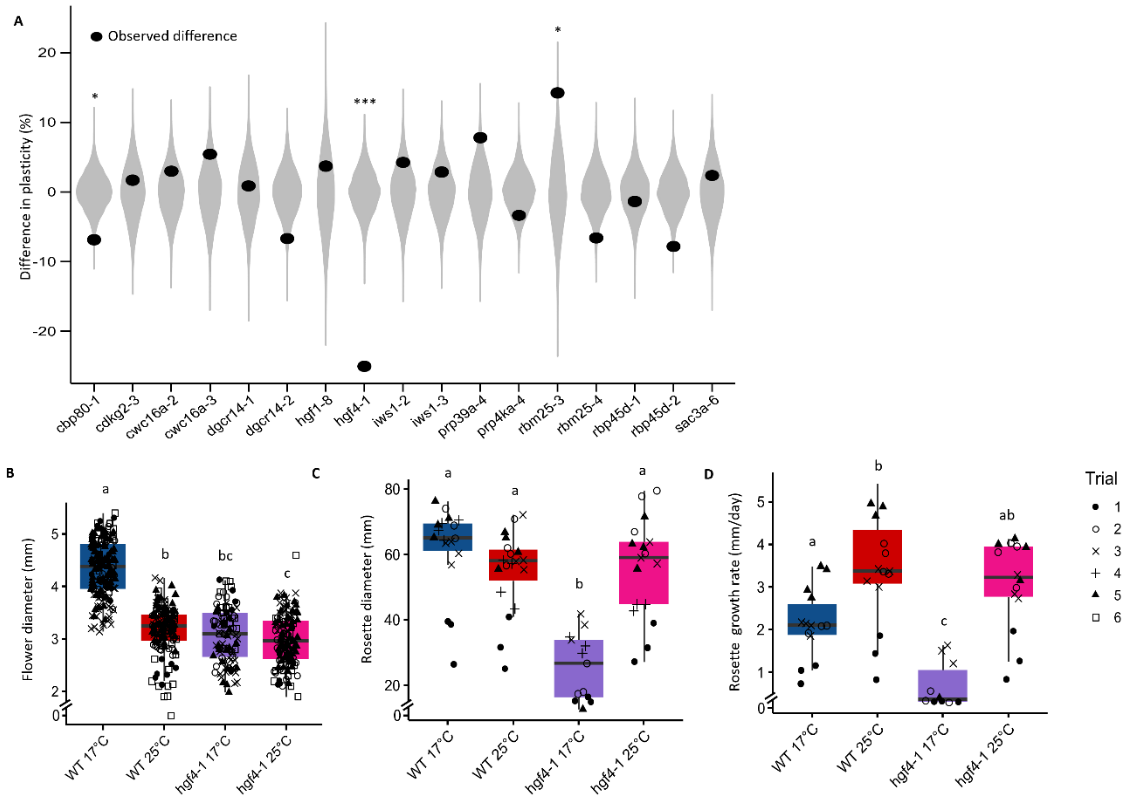

Supplementary Figure 1. **Comparison of temperature-mediated *hgf4-1* and WT plasticities across six trials.** A) Percentage of difference in plasticity in response to temperature in mutants compared to WT. Bonferroni corrected empirical p-values from permutation test with 5,000 iterations. * p-value < 0.05, *** p-value < 0.001. B) Flower diameters, C) rosette diameters and D) rosette growth rates of WT and *hgf4-1* plants grown at 17 and 25 °C in six independent growth trials. In B-D: Tukey's HSD, Bonferroni corrected p-value < 0.05. Groups that share a letter are not significantly different.

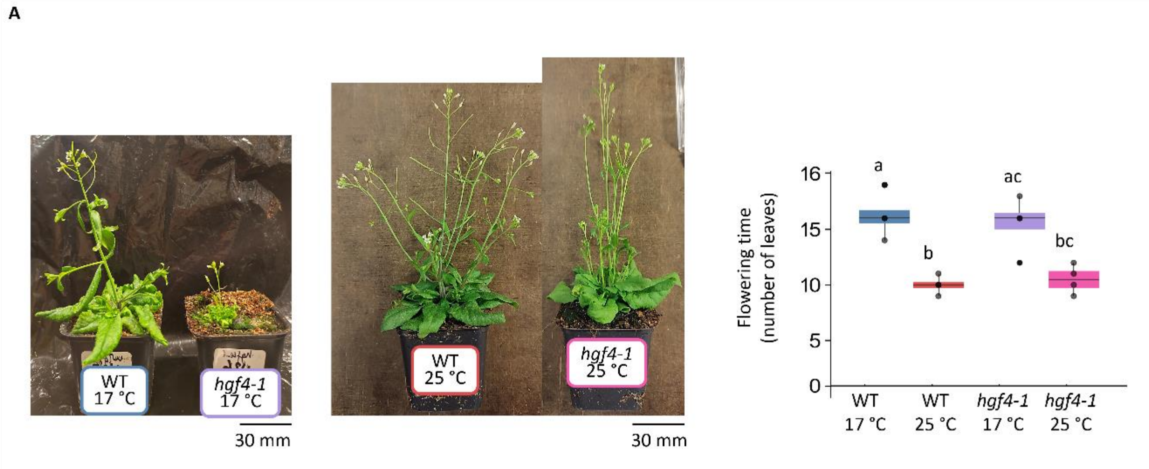

Supplementary Figure 2. Photographs of WT and *hgf4-1* plants grown at 17 and 25 °C. Inflorescences of WT and *hgf4-1* plants grown at 17 and 25 °C. Flowering time was quantified as number of leaves, n=4. Tukey's HSD, Bonferroni corrected p-value < 0.05. Groups that share a letter are not significantly different.

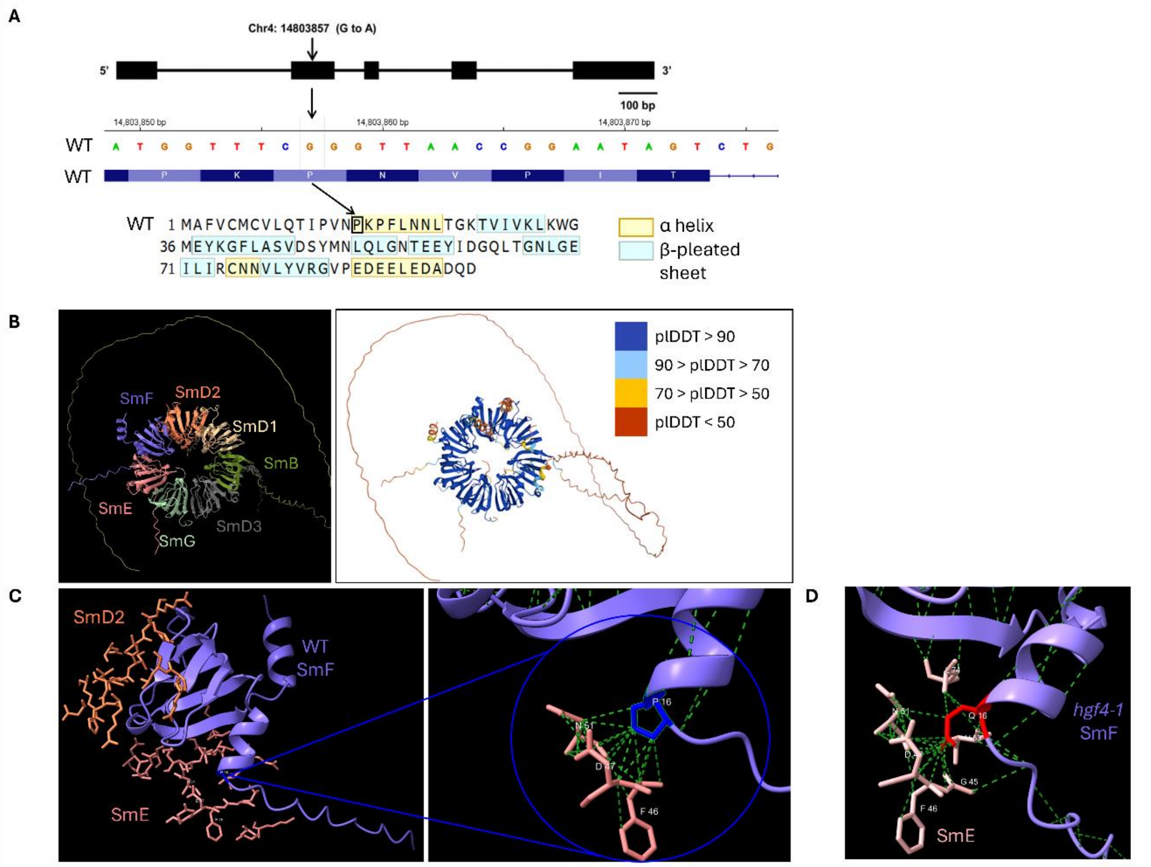

Supplementary Figure 3. ***In sillico*** **modelling of Sm proteins.** A) A schematic presentation showing the location of the EMS mutated SNP in *hgf4-1* reported in Kanno et al. (2020). The nucelotide and protein sequences provided are in the wild-type (WT) Columbia-0 background. B) Predicted heptamer protein structure with WT SmF and the six other Sm proteins. Predicted local distance difference test (plDDT) is a per-residue measure of local confidence used to assess the accuracy of the model. C) WT SmF showed predicted contact residues to SmE and SmD2. The location of the P16 non-synonymus mutation site is highlighted in dark blue and shows three contact residues between SmF P16 and SmE F46, D47 and N51. Atom interactions (< 4.00 Å) are shown by the dashed green lines. D) *hgf4-1* SmF protein was modelled to acquire three additional amino acid residue contact sites by showing atom interactions between SmF Q16, highlighted in red, and SmE G45, F46, D47, N51 V53 and L74.

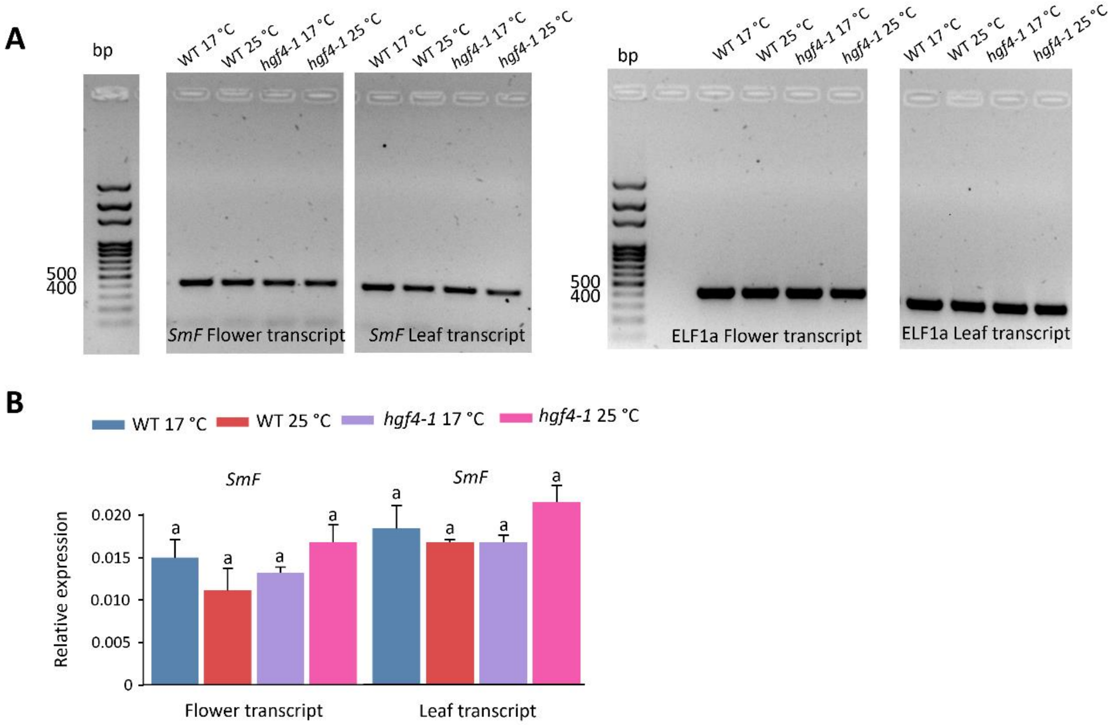

Supplementary Figure 4. *SmF* expression in WT and *hgf4-1* plants grown at 17 and 25 °C A) semi-quantitative PCR of *SmF* and reference *ELF1a* genes. B) *SmF* gene expression levels from flower and leaf RNA were also quantified via RT-qPCR. Relative expression levels were compared via Tukey's HSD with Bonferroni correction, n=6. Error bars show standard error.

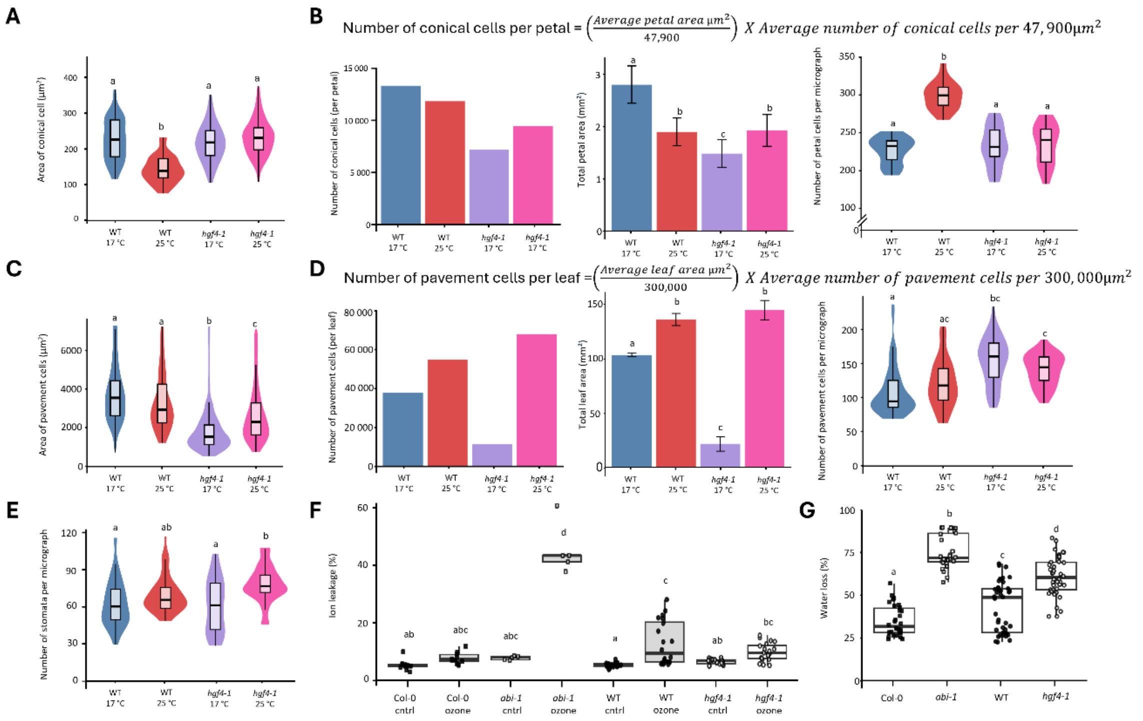

Supplementary Figure 5. Microscopic and physiological analysis of petal and leaf cellsin WT and *hgf4-1* plants grown at 17 and 25 °C. Micrographs, covering 47 900 µm^2 of petal epidermis, were taken from petal samples collected from open flowers covering the top and middle zones of the petal. A) The average largest petal conical cell area in 47 900 µm^2 sections of petal tissue from WT and *hgf4-1* open flowers. n=60. B) Estimated number of petal conical cells per whole petal. The total area of individual petals was measured, n=16, and the total petal conical cell numbers per micrograph were enumerated , n= 20. Error bars show standard error. Micrographs, covering 300 000 µm^2 of adaxial leaf epidermis were taken from leaf sections covering the top middle and bottom zones of the leaf. C) Area of largest pavement cell in 300 000 µm^2 sections of leaves from WT and *hgf4-1* plants. n=144. D) Estimated number of pavement cells per whole leaf of WT and *hgf4-1* plants grown at 17 and 25 °C. The total area of individual leaves, n=3, and total number of pavement cells per micrograph, n= 48. Error bars show standard error. E) Number of stomatal cells in 300 000 µm^2 epidermal adaxial sections of leaves. n=144. F) Cell death, quantified as percentage of ion leakage, Col-0 (n=20), *abi1-1* in Col-0 background (n=10), WT-GFP (n=40) and *hgf4-1* in the background of WT-GFP (n=39) plants subjected to 450 nl L^{-1} ozone. G) Percentage of water loss from cut leaves of Col-0 (n=30), *abi-1* (n=30), WT-GFP (n=40) and *hgf4-1* (n=40) after 2 hours drying at room temperature. In A-G: Tukey's HSD, Bonferroni corrected p-values < 0.05. Groups that share a letter are not significantly different.

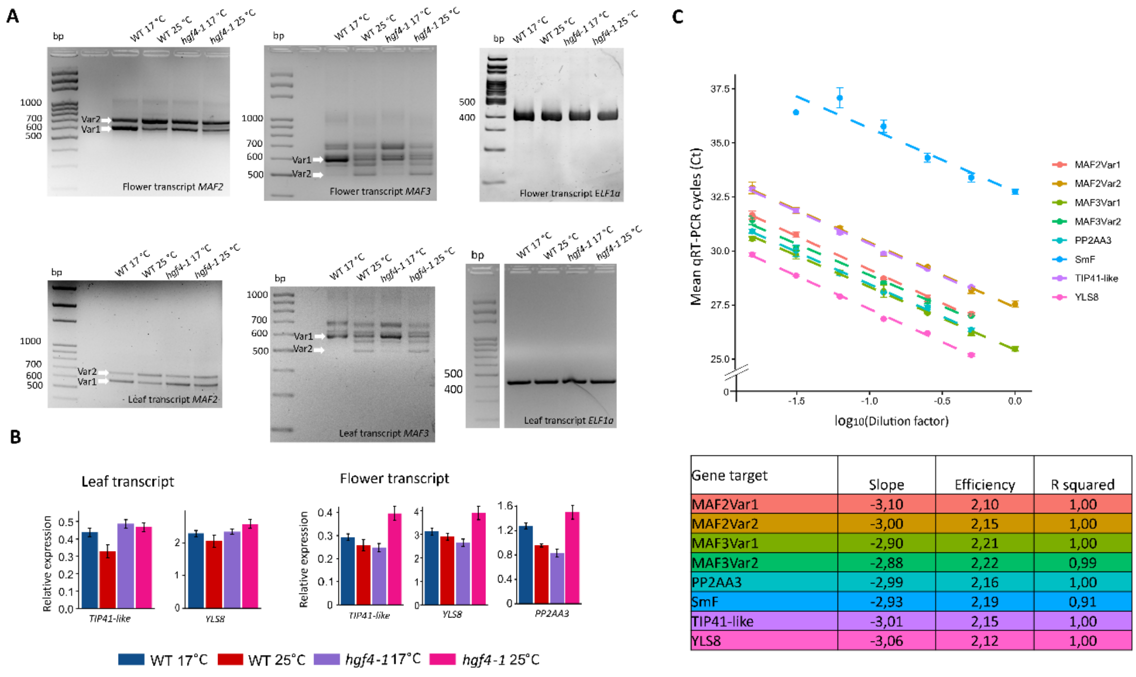

Gene target	Slope	Efficiency	R squared
MAF2Var1	-3,10	2,10	1,00
MAF2Var2	-3,00	2,15	1,00
MAF3Var1	-2,90	2,21	1,00
MAF3Var2	-2,88	2,22	0,99
PP2AA3	-2,99	2,16	1,00
SmF	-2,93	2,19	0,91
TIP41-like	-3,01	2,15	1,00
YLS8	-3,06	2,12	1,00

Supplementary Figure 6. Splice variants and gene expression profiling of *MAF2* and *MAF3*. A) *MAF2* and *MAF3* were alternatively spliced in WT and *hgf4-1* flowers and leaves in response to temperature. *ELF1a* was used as a reference gene to validate cDNA quality and quantity. B) *TIP41-like YLS8* and *PP2AA3* were used as reference genes for normalization of target genes in leaf and flower transcripts. Error bars show standard error. C) Standard curves used to calculate amplification efficiency of gene targets used in relative expression calculation for quantitative RT-PCR.

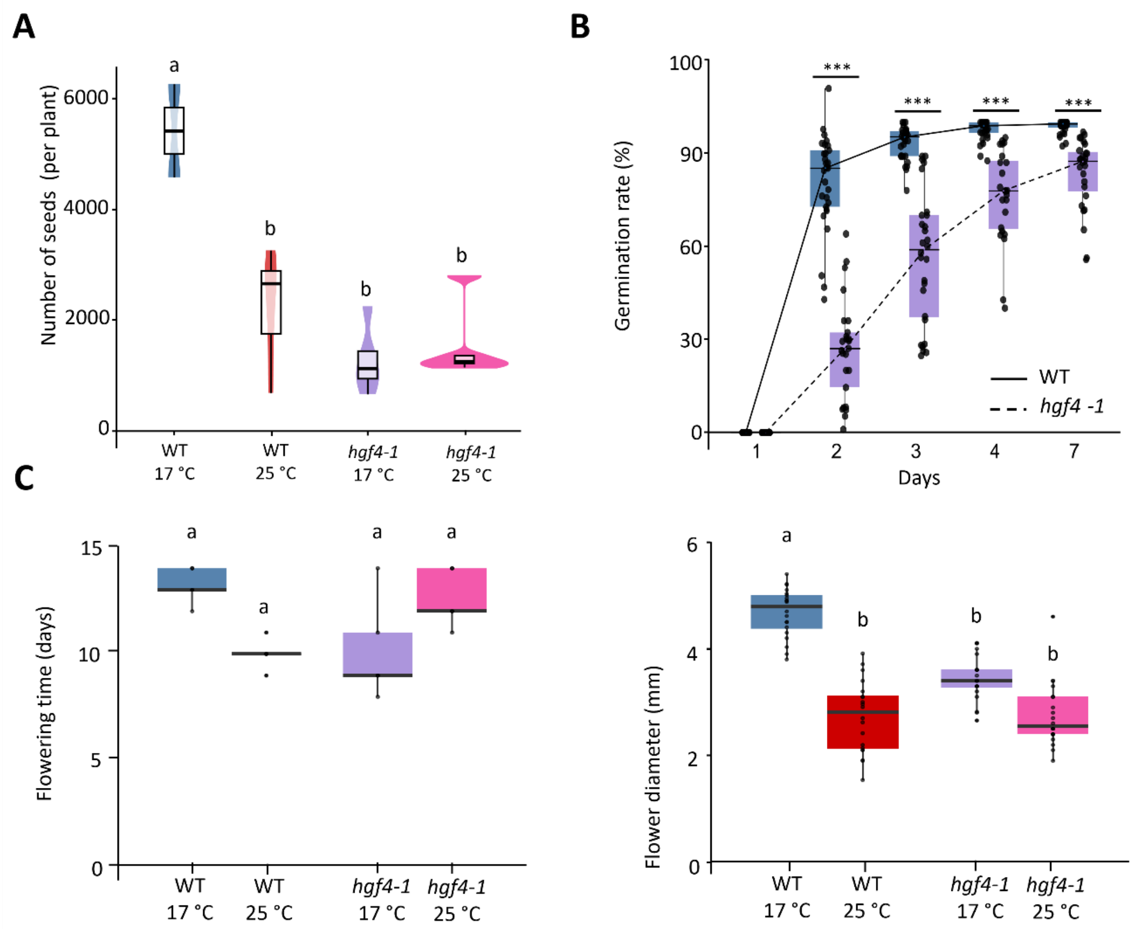

Supplementary Figure 7. Fitness estimates for WT and *hgf4-1* plants. A) Total number of seeds collected from a single plant grown at 17 and 25 °C. n=5. B) Germination rates of seeds collected from WT and *hgf4-1* plants grown at constant 17 °C, over 7 days. C) Flowering time, n=4 and flower size, n=24 for plants used to assess fitness was in line with what was seen earlier, *i.e.*, no difference in flowering time between WT and *hgf4-1*, and small robust flower diameters seen in *hgf4-1*. In A and C: Tukey's HSD, Bonferroni corrected p-value < 0.05, Groups that share a letter are not significantly different. In B: Unpaired t-test, n=28 WT n=32 *hgf4-1*. Bonferroni correction applied. *** p-value < 0.001.

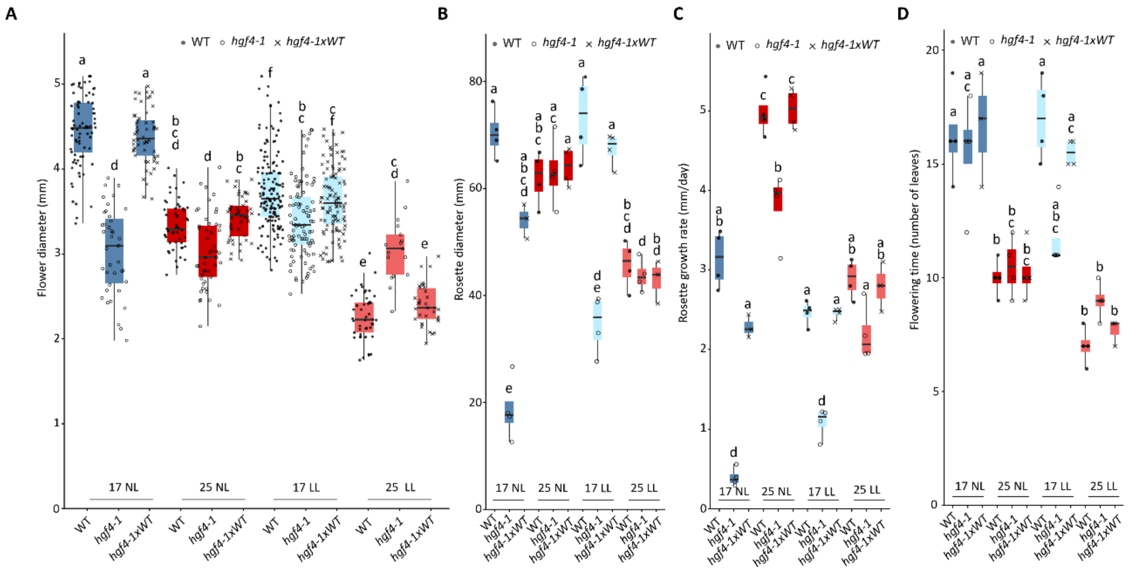

Supplementary Figure 8. Characterization of light-mediated flower size plasticity in *hgf4-1* grownat 17 and 25 °C. A) Flower diameters of WT, *hgf4-1* and *hgf4-1* back-crossed to WT (*hgf4-1* x WT) grown at 17 or 25 °C and under normal light (NL) 180 µmol m^{-2} s^{-1} or low light (LL) 45 µmol m^{-2} s^{-1}. Tukey's HSD with Bonferroni correction compared mean flower diameters, p-value < 0.05. Groups that share a letter are not significantly different. B) Rosette diameter, C) flowering time and D) rosette growth rate were also measured from the same plants. Tukey's HSD, n=4. p-value < 0.05.

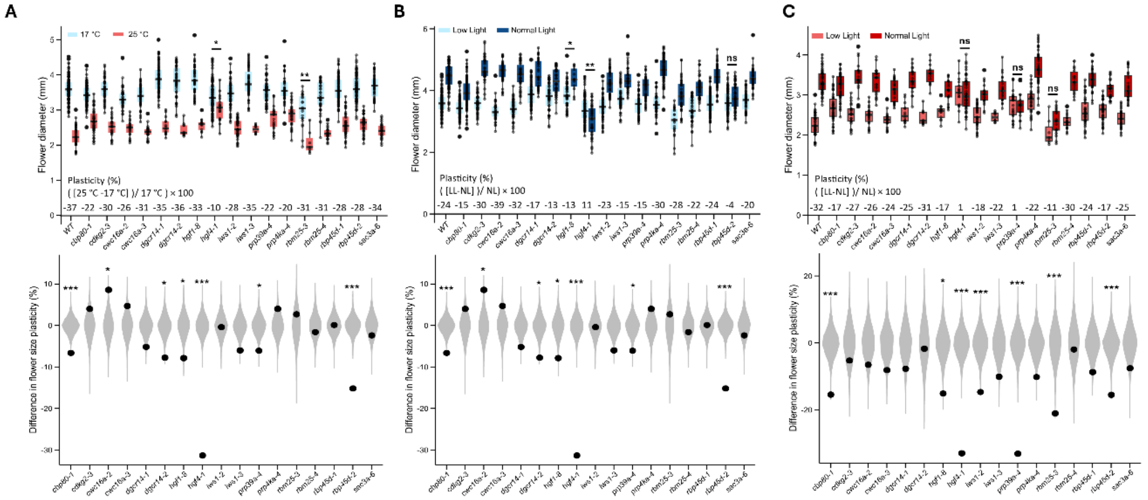

Supplementary Figure 9. Mean flower diameters and flower size plasticities of 17 spliceosome-related mutants in response to temperature and light-intensity. Difference in plasticity of the mutants compared to WT is shown as black dot. Permuted null distribution of differences in flower size plasticity in response to temperature and light between mutants and WT is shown by grey violin plot. A) Plants grown under constant LL either at 17 or 25 °C. Flower size plasticity is presented as a change in flower diameter from 17 to 25 °C at LL. B) Plants grown at constant 17 °C under either LL or NL. Flower size plasticity is presented as a change in flower diameter from NL to LL at 17 °C. C) Plants grown at constant 25 °C under either LL or NL. Flower size plasticity is presented as a change in flower diameter from NL to LL at 25 °C. In A-C Permutation test, Bonferroni corrected p-values, * p-value < 0.05, ** p-value < 0.01, *** p-value < 0.001. ns= not significant. NL= normal light, 180 µM m-2 s-1, LL = low light, 45 µM m-2 s-1.

	17 °C NL	25 °C NL	17 °C LL	25 °C LL
Flowering time	=	=	=	=
Flower diameter	-	-	-	+
MAF2var1 *MAF3var1*	-	=	NM	NM
Cell division	-	-	NM	NM
Cell expansion	=	+	NM	NM

	17 °C NL	25 °C NL	17 °C LL	25 °C LL
Rosette diameter	-	=	-	=
PR1 *PAD3*	+	=	NM	NM
SAG13 *TAT3*	+	+	NM	NM
Cell division	-	+	NM	NM
Cell expansion	-	-	NM	NM

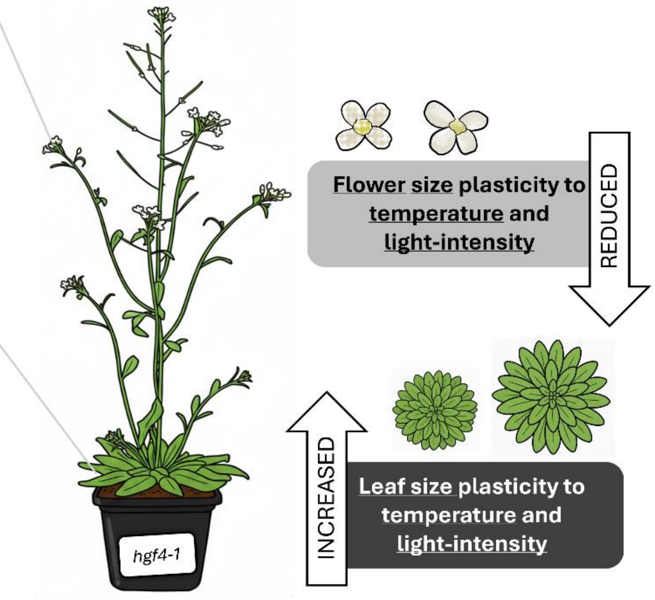

Supplementary Figure 10. Summary of the key traits in *SmF* mutant *hgf4-1* that were associated with differences in temperature and light-mediated growth plasticity. Tables show phenotypic differences between wild-type and *hgf4-1* at different temperatures, 17 and 25 °C, and under different light intensities low light (LL) = 45 µmol m^{-2} s^{-1} and normal light (NL) =180 m^{-2} s^{-1} . Table key: NM not measured, – less than WT, = same as WT, + more than WT.

SI References

T. Kanno *et al.*, A Collection of Pre-mRNA Splicing Mutants in *Arabidopsis thaliana*. *G3 Genes|Genomes|Genetics* **10**, 1983-1996 (2020).

T. Kanno *et al.*, PRP4KA, a Putative Spliceosomal Protein Kinase, Is Important for Alternative Splicing and Development in. *Genetics* **210**, 1267-1285 (2018).

T. Kanno *et al.*, A Genetic Screen for Pre-mRNA Splicing Mutants of Identifies Putative U1 snRNP Components RBM25 and PRP39a. *Genetics* **207**, 1347-1359 (2017).

T. Kanno *et al.*, Identification of Coilin Mutants in a Screen for Enhanced Expression of an Alternatively Spliced Reporter Gene in. *Genetics* **203**, 1709-+ (2016).

T. Kanno, W. D. Lin, J. L. Fu, A. J. M. Matzke, M. Matzke, A genetic screen implicates a CWC16/Yju2/CCDC130 protein and SMU1 in alternative splicing in. *Rna* **23**, 1068-1079 (2017).
