# Supplementary material for: Spliceosomal Factor SmF Modulates Temperature‐Mediated Flower and Leaf Size Plasticity in Arabidopsis thaliana
Source: Plant Cell Environ. 2026 Jan 5;49(4):2024–7. doi: 10.1111/pce.70358 (PMC12976579; doi:10.1111/pce.70358)
Supplement: Supplementary file 2 — Table S1: Mutant lines used in this study. Table S2: Flower diameters and statistical testing of plant lines grown at constant 17 or 25°C. Plant lines were compared to WT at each temperature and also comparisons were made within one plant line across the two temperatures. (t‐test with Bonferroni correction, ***p‐value < 0.001. ***p‐value 0.01, *p‐value < 0.05, ns = not significant.). Table S3: Primers for the gene targets used in this study. [file PCE-49-2024-s001.docx]

**Supporting Tables for**

Spliceosomal factor *SmF* modulates temperature-mediated flower and leaf size plasticity in Arabidopsis thaliana

Gregory M. Andreou-Huotari^1^, Mikael Brosché^1^, Jan Hoffmann^1^, Zoran Nikoloski^2,3^ and Roosa A. E. Laitinen^1*^

^1^ Organismal and Evolutionary Biology Research Programme, Viikki Plant Science Centre, University of Helsinki, 00790 Helsinki, Finland

^2^ Systems Biology and Mathematical Modelling, Max Planck Institute of Molecular Plant Physiology, 14476 Potsdam, Germany

^3^ Bioinformatics Department, Institute of Biochemistry and Biology, University of Potsdam, 14476 Potsdam, Germany

*Corresponding author: Roosa Laitinen, Organismal and Evolutionary Biology Research Programme, Viikki Plant Science Centre, PO Box 65, FIN-00014 University of Helsinki, Tel. +358 (0) 29 4157787, E-mail: Roosa.Laitinen@Helsinki.fi

Paste corresponding author name here

Email: [Roosa.Laitinen@Helsinki.fi](mailto:Roosa.Laitinen@Helsinki.fi)

**This PDF file includes:**

Tables S1 to S3

Table S1. Mutant lines used in this study.

Table S2. Flower diameters and statistical testing of plant lines grown at constant 17 or 25 °C. Plant lines were compared to WT at each temperature and also comparisons were made within one plant line across the two temperatures. (t-test with Bonferroni correction, *** p-value < 0.001. *** p-value 0.01, * p-value < 0.05, ns = not significant.)

Table S3. Primers for the gene targets used in this study.
