# Supplementary material for: Spliceosomal Factor SmF Modulates Temperature‐Mediated Flower and Leaf Size Plasticity in Arabidopsis thaliana
Source: Plant Cell Environ. 2026 Jan 5;49(4):2024–7. doi: 10.1111/pce.70358 (PMC12976579; doi:10.1111/pce.70358)
Supplement: Supplementary file 3 — Supplemental Materials and Methods. [file PCE-49-2024-s002.pdf]

Materials and Methods for

Spliceosomal factor *SmF* modulates temperature-mediated flower and leaf size plasticity in *Arabidopsis thaliana*

Gregory M. Andreou-Huotari[1], Mikael Brosché[1], Jan Hoffmann[1], Zoran Nikoloski[2,3] and Roosa A. E. Laitinen[1*]

[1] Organismal and Evolutionary Biology Research Programme, Viikki Plant Science Centre, University of Helsinki, 00790 Helsinki, Finland

[2] Systems Biology and Mathematical Modelling, Max Planck Institute of Molecular Plant Physiology, 14476 Potsdam, Germany

[3] Bioinformatics Department, Institute of Biochemistry and Biology, University of Potsdam, 14476 Potsdam, German

*Corresponding author: Roosa Laitinen, Organismal and Evolutionary Biology Research Programme, Viikki Plant Science Centre, PO Box 65, FIN-00014 University of Helsinki, Tel. +358 (0) 29 4157787, E-mail: Roosa.Laitinen@Helsinki.fi

Paste corresponding author name here

Email: Roosa.Laitinen@Helsinki.fi

Plant material and growth conditions

Mutant and WT seeds were ordered from the European Arabidopsis Stock Centre (NASC, Table S1). The set of 17 mutants were chosen based on either hyperactive or reduced levels of GFP signal compared to the wild-type (WT) line, due to differential splice variant abundance that gives rise to increased, or decreased, translatable GFP mRNA respectively (Kanno *et al.*, 2016; Kanno *et al.*, 2017). Many of the mutants, including *hgf4-1* were validated using complementation (Kanno et al., 2017). Prior to sowing, the seeds were stratified over two nights in 0.01 % agar solution in the dark at 4 °C. The seeds were sown in 2:1 peat:vermiculite soil mixture in 6x6 cm pots and germinated in Percival growth chambers (CLF Plant Climatics) either at constant 17 or at 25 °C under long day (LD, 16hr day/ 8hr night) conditions with 60 % relative humidity. Under normal light (NL) conditions, the light intensity was set to 180 µmol m^{-2} s^{-1} and for testing under low light (LL) conditions, 45 µmol m^{-2} s^{-1} was applied. 2-4 biological replicates were assessed for each plant line. To lessen the effects of chamber microclimates, the pots were randomized within the trays, and the trays were rotated and placed into different positions within the chamber every second day. Growth trials with 17NL and 25NL were independently carried out a total of six times, over the course of the investigation, each with 2-4 biological replicates.

Phenotyping flower diameter, rosette diameter, and flowering time

For flower diameter (FD), after the third open flower emerged, flowers were picked from the main inflorescence and the resulting two flower diameters per flower was measured using ImageJ. For rosette diameter (RD) and rosette growth rate (RGR), photographs of trays were taken from above every two or three days, approximately three hours after the lights came on, until flowering. RD was measured at the onset of flowering, and RGR was calculated as the gradient of the line of rosette size over time flowering time (FT) was measured as number of leaves at the onset of flowering. Flower and leaf size plasticity were calculated by $\frac{\mu\text{ trait at }23°C - \mu\text{ trait at }17°C}{\mu\text{ trait at }17°C}$.

Protein modelling

Alphafold 3 (Abramson *et al.*, 2024) was used to model the Sm heptamer ring using protein sequences downloaded from Arabidopsis information resource (TAIR), namely SmF (AT4G30220.2), SmE (AT2G18740.1), SmD1 (AT3G07590.1), SmD2 (AT2G47640.1), SmD3 (AT1G76300.1), SmB (AT5G44500.1) and SmG (AT3G11500.1). Modelling was performed using five seeds each with five independent models for each prediction and the resulting protein structures were superimposed using ChimeraX matchmaking. The models were assessed via predicted template modeling (pTM) scores and interface predicted template modeling (ipTM) scores. These measures of accuracy are for the entire structure (Zhang & Skolnick, 2004; Xu & Zhang, 2010).

Fitness testing

To test fitness of WT and *hgf4-1* plant lines, quantified as seed number and germination, five biological replicates were grown as before (Methods, plant material and growth conditions) at constant 17 or at 25 °C under NL. FD and FT were measured as described earlier, and in line with the earlier investigation, i.e., *hgf4-1* plants showed significantly smaller FDs at 17 °C and no differences were seen in FT between hgf4-1 and WT (Figure S7). To equalize development, the plants were kept in the growth chambers until the tenth silique was > 10 mm and then moved the plant to greenhouse LD rooms. The watering was stopped after ten days to allow natural senescence and the maturation of the seeds. Fully dry seeds were harvested from the plants and 100 seeds were weighed. The single seed weight (SSW) was obtained by dividing the 100 seed weight by 100. The total number of seeds was quantified by dividing total seed weight by SSW. For the germination assay, 100 seeds from each of the five biological replicates grown at 17 °C, were plated onto 0.5xMS plates and grown in chambers (Panasonic MLR-352-PE) with 12h day and 12h night with 22 °C/day and 18 °C/ night. Germination plates performed with six technical replicates. Prior plating, seeds were surface sterilized (95% ethanol + 0.5 % Triton-X) and stratified at 4 °C under darkness for 2 days. First four days seed germination was counted daily at 9:00 and a final germination rate (a percentage of germinated seeds / total number of seeds) was quantified after seven days.

Ozone sensitivity and water loss assay

For ozone sensitivity testing, Col-0, *abi1-1* (originally a gift from Julian Schroder, and subjected to three rounds of back-crossing to Col-0 and known to be ozone sensitive (Merilo *et al.,* 2013), WT-GFP and *hgf4-1* plants were grown at short day (250 µmol m^{-2} s^{-1}, 12/12 h of light/dark and 23 °C/ 19 °C day/night) and then subjected to 450 nl L^{-1} ozone for 6 h. As the aim here was to test tolerance, we used higher doses of ozone than those normally used to test ozone sensitivity. Cell death was quantified from whole rosettes as ion leakage from control and ozone treated plants. Ions were measured 2 h after ozone treatment in 18 MΩ water with conductivity meter (Mettler Toledo GmbH, Greifensee, Switzerland) and then re-measured after overnight freezing to quantify total ion capacity in rosettes. Ion leakage was expressed as % ions released due to oxidative stress, like Overmyer *et al.* (2008). For the water loss assay Col-0, *abi1-1*, WT and *hgf4-1* plants were grown as for ozone testing, and 30-40 biological replicates, over 4 trials, were investigated by cutting 2-3 leaves per plant at 9:00am and immediately recording the gross weight. Leaves were then left abaxial side up under light for ca. 2 h and then re-weighed. Water loss was expressed via the decrease in weight as a percentage.

Microscopy

To investigate differences in leaf epidermis between WT and *hgf4-1*, four plants from each line were grown at constant at 17 and 25 °C under NL. When the plants reached the 8-10 leaf stage, the largest leaf pair was picked and de-stained overnight in 7:1 acetic acid:ethanol solution. The total leaf area was measured via photographs and the top, middle and bottom zones of the leaves were dissected from both sides of the main leaf vain and fixed to glass slides, abaxial side up, using Hoyer's medium. From each leaf zone, two micrographs were taken under 20 x magnification BIC using a DMLB Leica microscope. In total, 47 micrographs were assessed for each line, at each condition. The number of pavement and stomata cells were enumerated and three of the largest pavement cells in the 300 000 μm^2 area were measured. The total pavement cell number was approximated using the average total leaf area and the average number of pavement cells per micrograph enumerated (Figure S5D). For petal samples, five petals were assessed per line, per growth condition for conical cell number and area. The top and middle zones of each petal were investigated under 50 x magnification providing a 47 900 μm^2 area. The total conical cell number was approximated using average whole petal area and the average number of conical cells per micrograph enumerated (Figure S5B).

cDNA synthesis and analysis of splicing patterns

To investigate differences in splicing patterns in flowers and leaves, both semiquantitative and real-time quantitative RT-PCRs were used. WT and *hgf4-1* plant lines were prepared as before and grown at constant at 17 and 25 °C under NL. For transcript profiling in leaves, the youngest emerging leaf pairs were pooled from 4 independent plants at 8-10 leaf stage, in duplicate, providing two biological

replicates. Leaves were collected to liquid nitrogen three hours after the lights were turned on. For flower samples, two stage 12, according to Smyth *et al.* (1990), flower buds were pooled from 5 independent plants to liquid nitrogen, in duplicate, providing two biological replicates. Samples were ground using glass beads and RNA was isolated using TRIzol (Invitrogen, 15596026) according to the manufacturer's guidelines. The extracted nucleic acid was treated with DNAseI (Thermo fisher, 18047019), and 1 µg of RNA was used for cDNA synthesis using RevM Revertase (Klever lab, E-RT) according to the manufacturer's guidelines. For differential splicing patterns of *MAF2* and *MAF3,* real-time quantitative RT-PCR was used. Primers were designed to amplify cDNA from the first to the last exon. *ELF1a* was included as a reference for the cDNA (Supplementary Figure 6). Semi-quantitative PCR, visualising the different splice variants, was done using Phusion polymerase (Thermofisher, F530S, Table S3). Splice variants were visualised using 2.5% agarose gels and all splice variants 1 and 2 for both *MAF2* and *MAF3* were confirmed via Sanger sequencing. Primers were designed to distinguish splice variant 1 and 2 for both *MAF2* and *MAF3* (Figure 1B, Table S3), using real time quantitative PCR described below.

Gene expression analysis

For the synthesized leaf and flower cDNA, real-time qRT-PCR reactions were performed using 2X Fast Dye qPCR Mastermix, (Klever labs, M2-FD), on a Bio-Rad Opus CFX-384. *YLS8*, *PP2AA3* and *TIP41-like* (Stajner *et al.*, 2013) were used as housekeeping genes for normalisation, considering a standard deviation of ≤ 0.5 Ct as quality control. The two biological replicates for petals, and two for leaves, were tested via three technical replicates each for reaction. For each sample, the target gene levels were normalized, and relative expression was quantified according to the $X^{-\Delta Ct}$ method, where X = the primer amplification efficiency (Figure S6C, Table S3). For the defense gene transcript investigation, the following gene markers were chosen.*PR1* was selected as a common marker for salicylic acid (SA) mediated defense signaling (Gaffney *et al.*, 1993; van Loon *et al.*, 2006), while *PAD3* was chosen to represent camalexin synthesis involved in pathogen resistance and *PDF1.2* was chosen as a marker for jasmonic acid (JA) and ethylene-mediated defense signaling (Penninckx *et al.*, 1998; Zhou *et al.*, 1999; Robert-Seilaniantz *et al.*, 2011). In addition, we investigated *SAG13* as a leaf senescence marker induced by abiotic stress and reactive oxygen species (Miller *et al.*, 1999; Dhar *et al.*, 2020) and *TAT3,* as it is thought to play a role in redox regulation and reactive oxygen stress (Brosché & Kangasjärvi, 2012).

Statistical analysis

All statistical analyses were performed in R. Unpaired t-test with Bonferroni correction was used to test for significant differences in mean flower size between each of the 17 mutants and WT at each growth condition and to investigate the size of the flowers across the growth conditions in each plant

line. To detect statistical differences in flower size plasticity between mutants and WT, a permutation test using 5,000 iterations with Bonferroni correction was used. For comparison of all presented trait mean values between WT and *hgf4-1* a two-way ANOVA followed by Tukey's honest significant difference (HSD) tests and Bonferroni's correction was used. As water loss and ozone experiments were performed over several trials, a linear mixed model with trial as random effect was employed to compare mean water loss between WT and *hgf4-1.* Bonferroni correction was applied.

References for Materials and Methods

A. Robert-Seilaniantz, M. Grant, J. D. Jones, Hormone crosstalk in plant disease and defense: more than just jasmonate-salicylate antagonism. *Annu Rev Phytopathol* **49**, 317-343 (2011).

D. R. Smyth, J. L. Bowman, E. M. Meyerowitz, Early Flower Development in Arabidopsis. *Plant Cell* **2**, 755-767 (1990).

E. Merilo *et al.*, PYR/RCAR receptors contribute to ozone-, reduced air humidity-, darkness-, and CO2-induced stomatal regulation. *Plant Physiol* **162**, 1652-1668 (2013).

I. A. Penninckx, B. P. Thomma, A. Buchala, J. P. Metraux, W. F. Broekaert, Concomitant activation of jasmonate and ethylene response pathways is required for induction of a plant defensin gene in Arabidopsis. *Plant Cell* **10**, 2103-2113 (1998).

J. Abramson *et al.*, Accurate structure prediction of biomolecular interactions with AlphaFold 3 (vol 630, pg 493, 2024). *Nature* **636**, E4-E4 (2024).

J. D. Miller, R. N. Arteca, E. J. Pell, Senescence-associated gene expression during ozone-induced leaf senescence in Arabidopsis. *Plant Physiol* **120**, 1015-1023 (1999).

J. R. Xu, Y. Zhang, How significant is a protein structure similarity with TM-score=0.5? *Bioinformatics* **26**, 889-895 (2010).

K. Overmyer *et al.*, Complex phenotypic profiles leading to ozone sensitivity in Arabidopsis thaliana mutants. *Plant Cell Environ* **31**, 1237-1249 (2008).

L. C. van Loon, M. Rep, C. M. Pieterse, Significance of inducible defense-related proteins in infected plants. *Annu Rev Phytopathol* **44**, 135-162 (2006).

M. Brosché, J. Kangasjärvi, Low antioxidant concentrations impact on multiple signalling pathways in partly through NPR1. *J Exp Bot* **63**, 1849-1861 (2012).

N. Dhar *et al.*, The Arabidopsis SENESCENCE-ASSOCIATED GENE 13 Regulates Dark-Induced Senescence and Plays Contrasting Roles in Defense Against Bacterial and Fungal Pathogens. *Mol Plant Microbe In* **33**, 754-766 (2020).

N. Stajner, S. Cregeen, B. Javornik, Evaluation of Reference Genes for RT-qPCR Expression Studies in Hop (L.) during Infection with Vascular Pathogen-. *Plos One* **8** (2013).

N. Zhou, T. L. Tootle, J. Glazebrook, Arabidopsis PAD3, a gene required for camalexin biosynthesis, encodes a putative cytochrome P450 monooxygenase. *Plant Cell* **11**, 2419-2428 (1999).

Y. Zhang, J. Skolnick, Scoring function for automated assessment of protein structure template quality. *Proteins* **57**, 702-710 (2004).
